# Supplementary material for: Dear admission committee…: Which moves in application essays predict student master grades?
Source: PLoS One. 2024 Jun 28;19(6):e0304394. doi: 10.1371/journal.pone.0304394 (PMC11213304; doi:10.1371/journal.pone.0304394)
Supplement: S1 File — (DOCX) [file pone.0304394.s001.docx]

Table 1: Full analysis betaregressions

| **Move probabilities** | **Master specific (1)** | **Research skills (2)** | **Prior education (3)** | **Societal Impact (4)** | **Interest to learn (5)** | **City & University (6)** | **Extracurricular (7)** |
| --- | --- | --- | --- | --- | --- | --- | --- |
|  | | | | | | | |
| **Word count application essay** | -0.0004^***^ | -0.0004^**^ | -0.001^***^ | 0.0003^*^ | 0.0001 | -0.001^***^ | -0.0003 |
|  |  |  |  |  |  |  |  |
| **Average bachelor grade** | 0.038 | 0.106^**^ | -0.005 | 0.053 | -0.173^***^ | 0.055 | -0.057 |
|  |  |  |  |  |  |  |  |
| **Gender (M = 1, F = 2)** | 0.050 | -0.146^**^ | 0.041 | 0.026 | 0.079 | 0.128^**^ | 0.082 |
|  |  |  |  |  |  |  |  |
| **Age** | 0.009 | -0.006 | -0.002 | 0.042^***^ | -0.005 | 0.028^**^ | -0.003 |
|  |  |  |  |  |  |  |  |
| **International background: Africa** | -0.206 | 0.093 | -0.441 | 0.118 | -0.215 | 0.891^***^ | 0.076 |
| **International background: Asia** | -0.168^*^ | 1.490^***^ | -0.196^**^ | 0.320^***^ | -0.156 | 0.780^***^ | -0.004 |
|  |  |  |  |  |  |  |  |
| **International background: Central America** | 0.218 | -0.432 | -0.392 | 0.761^***^ | -0.007 | 0.217 | 0.213 |
|  |  |  |  |  |  |  |  |
| **International background: Middle-East** | -0.372 | 0.465 | -0.266 | 0.447^*^ | -0.077 | 0.269 | -0.360 |
|  |  |  |  |  |  |  |  |
| **International background: North America** | 0.113 | 0.410^**^ | -0.349^**^ | 1.217^***^ | -0.168 | 0.397^**^ | -0.309 |
|  |  |  |  |  |  |  |  |
| **International background: South America** | -0.117 | 0.020 | -0.214 | 0.667^***^ | -0.373^**^ | 0.375^**^ | -0.223 |
|  |  |  |  |  |  |  |  |
| **Master program: Energy sciences** | 1.317^***^ | -0.169 | 0.444^***^ | 0.429^***^ | 0.316^**^ | 0.440^***^ | 0.454^***^ |
| **Master program: Earth, structure and dynamics** | 1.467^***^ | -0.060 | 0.345^***^ | 0.150 | 0.271^**^ | 0.331^**^ | 0.276^*^ |
| **Master program: Earth, surface and water** | 1.374^***^ | 0.018 | 0.204^*^ | 0.128 | 0.163 | 0.429^***^ | 0.120 |
| **Master program: Graphical information management and applications** | 2.912^***^ | -0.016 | 0.170 | 0.155 | 0.436^***^ | 0.560^***^ | 0.524^***^ |
| **Master program: Water science and management** | 0.013 | 0.002 | 0.285^**^ | 0.745^***^ | 0.530^***^ | 0.425^**^ | 0.383^**^ |
| **Master program: Human geography and planning** | 1.670^***^ | 0.446^**^ | 0.444^***^ | 0.405^**^ | 0.469^***^ | 0.309 | 0.396^**^ |
| **Master program: Marine sciences** | 1.290^***^ | 0.144 | 0.275^**^ | 0.344^**^ | 0.593^***^ | 0.392^**^ | 0.345^**^ |
| **Master program: Innovation sciences** | 1.176^***^ | -0.061 | 0.570^***^ | 0.241 | 0.263^*^ | 0.326^*^ | 0.378^**^ |
| **Master program: Sustainable business and innovation** | 1.427^***^ | -0.082 | 0.459^***^ | 0.677^***^ | 0.384^***^ | 0.448^***^ | 0.801^***^ |
| **Master program: Sustainable development** | 1.334^***^ | 0.103 | 0.296^**^ | 0.848^***^ | 0.521^***^ | 0.449^***^ | 0.513^***^ |
|  |  |  |  |  |  |  |  |
| **Constant** | -2.812^***^ | -2.984^***^ | -0.400 | -3.576^***^ | -0.148 | -2.647^***^ | -2.368^***^ |
|  |  |  |  |  |  |  |  |
|  | | | | | | | |
| **Observations** | 823 | 823 | 823 | 823 | 823 | 823 | 823 |
| **Pseudo-R^2^** | 0.514 | 0.208 | 0.081 | 0.317 | 0.072 | 0.120 | 0.085 |
| **Log Likelihood** | 617.697 | 1,400.619 | 387.771 | 836.172 | 524.043 | 507.487 | 1,284.320 |

|  |  | | | |
| --- | --- | --- | --- | --- |
| Dependent variable | Average master grade | | First year average master grade | |
| Model | (1) | (2) | (3) | (4) |
|  | | | | |
| Master specific |  | 0.453^**^ |  | 0.415^*^ |
|  |  |  |  |  |
| Research skills |  | -1.611^***^ |  | -1.903^***^ |
|  |  |  |  |  |
| Prior education |  | 0.304 |  | 0.273 |
|  |  |  |  |  |
| Societal impact |  | -0.684^**^ |  | -0.690^**^ |
|  |  |  |  |  |
| Interest to learn |  | 0.587^**^ |  | 0.733^**^ |
|  |  |  |  |  |
| City and University |  | -0.884^***^ |  | -0.905^***^ |
|  |  |  |  |  |
| Extracurricular |  | -0.232 |  | 0.354 |
|  |  |  |  |  |
| Word count application essay | 0.0003^***^ | 0.0003^***^ | 0.0003^***^ | 0.0003^**^ |
|  |  |  |  |  |
| Average bachelor grade | 0.331^***^ | 0.347^***^ | 0.367^***^ | 0.388^***^ |
|  |  |  |  |  |
| Gender (1= male, 2 = female) | 0.066^**^ | 0.061^**^ | 0.051 | 0.044 |
|  |  |  |  |  |
| Age | -0.0004 | 0.004 | -0.006 | -0.001 |
|  |  |  |  |  |
| International background: Africa | -0.678^***^ | -0.535^***^ | -0.749^***^ | -0.593^***^ |
| International background: Asia | -0.590^***^ | -0.378^***^ | -0.619^***^ | -0.380^***^ |
|  |  |  |  |  |
| International background: Central America | -0.853^***^ | -0.793^***^ | -0.924^***^ | -0.863^***^ |
|  |  |  |  |  |
| International background: Middle-East | -0.229 | -0.139 | -0.121 | -0.020 |
|  |  |  |  |  |
| International background: North America | -0.284^***^ | -0.161 | -0.240^**^ | -0.103 |
|  |  |  |  |  |
| International background: South America | -0.288^***^ | -0.184^*^ | -0.337^***^ | -0.225^**^ |
|  |  |  |  |  |
| Master program: Energy sciences | -0.039 | -0.081 | 0.120 | 0.072 |
|  |  |  |  |  |
| Master program: Earth, structure and dynamics | 0.010 | -0.056 | 0.062 | -0.001 |
|  |  |  |  |  |
| Master program: Earth, surface and water | 0.008 | -0.034 | 0.160^*^ | 0.122 |
|  |  |  |  |  |
| Master program: Graphical information management and applications | -0.161^*^ | -0.332^***^ | -0.124 | -0.293^**^ |
|  |  |  |  |  |
| Master program: Water science and management | -0.237^**^ | -0.200^**^ | 0.108 | 0.136 |
|  |  |  |  |  |
| Master program: Human geography and planning | 0.081 | 0.011 | 0.313^***^ | 0.241^**^ |
|  |  |  |  |  |
| Master program: Marine sciences | -0.170^*^ | -0.210^**^ | -0.150 | -0.194^**^ |
|  |  |  |  |  |
| Master program: Innovation sciences | -0.357^***^ | -0.410^***^ | -0.131 | -0.187^*^ |
|  |  |  |  |  |
| Master program: Sustainable business and innovation | -0.340^***^ | -0.366^***^ | -0.080 | -0.118 |
|  |  |  |  |  |
| Master program: Sustainable development | -0.159^**^ | -0.162^*^ | 0.056 | 0.048 |
|  |  |  |  |  |
| Constant | 5.179^***^ | 4.946^***^ | 4.791^***^ | 4.526^***^ |
|  |  |  |  |  |
|  | | | | |
| Observations | 823 | 823 | 820 | 820 |
| Adjusted R^2^ | 0.289 | 0.312 | 0.296 | 0.321 |
| Residual Std. Error | 0.416 (df = 802) | 0.409 (df = 795) | 0.442 (df = 799) | 0.434 (df = 792) |
| F Statistic | 17.734^***^ (df = 20; 802) | 14.832^***^ (df = 27; 795) | 18.224^***^ (df = 20; 799) | 15.315^***^ (df = 27; 792) |
|  | | | | |

Table 2: Full analysis OLS

^*^p^**^p^***^p<0.01
