# Supplementary material for: Dear admission committee…: Which moves in application essays predict student master grades?
Source: PLoS One. 2024 Jun 28;19(6):e0304394. doi: 10.1371/journal.pone.0304394 (PMC11213304; doi:10.1371/journal.pone.0304394)
Supplement: Attachment — Submitted filename: Response to Reviewers.docx [file pone.0304394.s002.docx]

Dear editor, dear reviewers,

We are grateful for the positive reception of the paper by both reviewers and the editors. Please find below how we addressed each comment.

**Comments from Editor**

You should give special emphasis on the references as more recent research should be given emphasis**.**

**We put extra emphasis on the more recent references by changing the order of certain sentences in the introduction. Furthermore, we expanded our description of the most relevant quantitative and recent papers which our paper builds on. We hope this satisfies the editors expectations.**

Methods need to be written in more detailed as which Masters' programs were included and why?

**We have now added a description of the faculty, a the motivation for the programs that we included, and the names of the programs.**

Format of the tables need to be improved.

**We updated to formatting of the tables to comply with the journals guidelines.**

**Comments from Reviewer 1:**

1.Looking at the content, including the analysis and conclusions of the study, I suggest the re-consideration of the study’s title or using the word "what are" in place of "how" in the study title since the study deployed quantitative analysis techniques to look at the topics in application essays and their correlations and prediction of student success. ( Additional reference to Line 558; the study’s aim)

**We agree with reviewer that how is not the best word in the title. Hence, we went for: “Dear admission committee…: Which moves in application essays predict student master grades?” We also opted to use the word ‘moves’ instead of ‘topics’ in the title, since this better reflects the contents of the paper**

2.Please consider reading the manuscript for some perceived minor grammatical mistakes. Example: The use of the word "motivate" in line 400 seems not to be appropriate**. (actie Frank)**

**We understand how this can lead to confusion. We changed the sentence to: *“After all, students write about master specific term to motivate how passionate they are for content of the master.”*** **Further, we read through the manuscript and made several other minor adjustments, and spelling corrections (see manuscript.)**

3.I suggest syntax interruptions be minimised throughout the paper for easy comprehension. For instance, the sentence "Furthermore, every international background, except "international background: Africa," has a more significant positive relationship compared with our reference category, Europe. (Line 437)" could be transformed into "Furthermore, every international background has a more significant positive relationship compared with our reference category, Europe, except "Africa."

**We followed this suggestion, and changed this on all occasions.**

**4. Students with a relatively low bachelor’s grade and unsuitable bachelor’s degree “would probably write little about their intrinsic motivation”. (Line 616-627) Any literature to back this strong claim?**

**This is a very specific claim that was not found in the scarce literature on motivation letters. In the end, we decided to let the data speak. We compared the data that we have about rejected students (which we could not include in our analysis, because we have no master grades for this group). Based on this, we found no indications for admission bias:**

***“However, our data for rejected students shows that there is hardly any correlation between the probability to use any of the relevant moves in the application essay, and the average bachelor grade (Pearsons r = -0.06, n=348), and the suitability of the bachelor program (Pearsons r = -0.08, n=375). These correlations hardly differ from those of admitted students (average bachelor grade: Pearson’s r = -0.04, n=1582 and suitability Pearsons r = -0.02, n=2755). In a univariate binary logistic regression, we did find a positive significant relationship between the probability of using relevant moves and being admitted or not (odds ratio=48.99). This result is theoretically expected as the use of the relevant moves is expected to contribute to study success. Based on these results, we see little indication of admission bias.*”**

5.For transparency purposes, any analysis carried and discussed or mentioned in the paper should be made available for readers (as a supporting document). example: "We controlled for these disciplines with our education programme."dummies, and found they do not significantly influence our main findings. **"(actie Timon)**

**This is very good point by the reviewer. We followed this suggestion and made this analyses available in a supporting document. We did this for both the betaregressions and the OLS.**

6.We are told continents were used because the sample size was small for some regional and sub-regional groups. It is appropriate for us to know the total number of participants from each continent and whether all were adequate for the analysis carried. I suggest a table for the characteristics of the participants used, including sex, continent, age, type of master’s programme, and others available. Such information might be relevant for making appropriate inferences from the study, e.g., the deviations of African students from some of the study findings.

**We included a table with means and standard deviations for the numerical variables, and distributions and standard deviations for the categorical variables as well as a correlation matrix for all numeric variables in the methodology.**

7. I doubt if the tables meet the PlosOne specifications.

**We updated to formatting of the tables to comply with the journals guidelines.**
