# Supplementary material for: Dear admission committee…: Which moves in application essays predict student master grades?
Source: PLoS One. 2024 Jun 28;19(6):e0304394. doi: 10.1371/journal.pone.0304394 (PMC11213304; doi:10.1371/journal.pone.0304394)
Supplement: Attachment — Submitted filename: Response to Reviewers.docx [file pone.0304394.s003.docx]

Dear editor, dear reviewers,

We are grateful for the positive reception of the paper by both reviewers and the editors. Please find below how we addressed each comment.

Reviewer #1:

1. It is recommended that authors explicitly state the study design under Section 3 (Methods).

**We would like to thank reviewer 1 for this useful suggestion. We added the study design in the beginning of Section 3, in line 247.**

2. Considering gender as binary is becoming outdated and unacceptable. Gender, as a social construct, is not binary (male or female). Perhaps sex, a biological construct often measured as binary, could be used in place of gender in this study.

**This is a very good point by reviewer 1. We changes all references to ‘gender’ in the main text to ‘sex’, and added an explanatory footnote on page 16.**

3. Please review the sentence “We connected... master” (lines 422–424) and similar ones. Conduct a thorough proofread of the entire work to ensure that all sentences are complete, easy to comprehend, and in the appropriate tense.

**We carefully revised Section 4 of the manuscript and corrected any incorrect tenses. We now use the past tense when referring to activities that occurred in the past, and the present tense when making factual statements. We conducted a proofread of the manuscript, and enlisted a professional editor to do the same in an earlier stage. Based on this, we are confident that the work is easy to comprehend.**
